# Sirtuin 1 rs7069102 polymorphism is associated with diabetic nephropathy in patients with type 2 diabetes mellitus

**DOI:** 10.17305/bjbms.2020.5368

**Published:** 2021-10

**Authors:** Jernej Letonja, Matej Završnik, Jana Makuc, Maja Šeruga, Ana Peterlin, Ines Cilenšek, Daniel Petrovič

**Affiliations:** 1 Institute of Histology and Embryology, Faculty of Medicine, University of Ljubljana, Ljubljana, Slovenia; 2 Department for Diabetes and Metabolic Diseases, Clinic for Internal Medicine, University Medical Centre Maribor, Maribor, Slovenia; 3 Department of Internal Medicine, General Hospital Slovenj Gradec, Slovenj Gradec, Slovenia; 4 Department of Internal Medicine, General Hospital Murska Sobota, Murska Sobota, Slovenia

**Keywords:** SIRT1, rs7069102, diabetic nephropathy, type 2 diabetes mellitus, association study

## Abstract

The global prevalence for diabetes mellitus nearly doubled from 4.7% in 1980 to 8.5% in 2014. Sirtuin 1 (SIRT1) is an NAD+-dependent deacetylase that is expressed in a variety of tissues. It modifies proteins that participate in DNA repair, stress, and inflammatory response. The aim of the study was to investigate the relationship between SIRT1 rs7069102 polymorphism and diabetic nephropathy (DN) in patients with type 2 diabetes mellitus (T2DM). In our retrospective association study, we included 724 Slovene (Caucasian) patients who have had T2DM for at least 10 years. We classified the participants into two groups, the first group was comprised of 301 patients with DN, and the second (control) group was comprised of 423 patients without DN. We analyzed the rs7069102 polymorphism using StepOne real-time polymerase chain reaction (PCR) System and TaqMan SNP Genotyping Assay. We found a statistically significant difference in the distribution of rs7069102 genotypes and alleles between the two groups. We used logistic regression analysis and adjusted for systolic pressure, arterial hypertension (AH), duration of AH, triglycerides, the value of HbA1c, carotid disease, diabetic foot, and diabetic retinopathy. Furthermore, we discovered that patients with the CC genotype are significantly more likely to develop DN according to both the codominant (odds ratio [OR] = 1.94; 95% confidence interval [CI] = 1.09-3.45; p = 0.02) and recessive (OR = 2.39; 95% CI = 1.12-5.08; p = 0.02) models of inheritance. We found a significant association between the SIRT1 rs7069102 polymorphism and DN in T2DM. We speculate that SIRT1 rs7069102 might be an interesting marker of DN.

## INTRODUCTION

The global prevalence for diabetes mellitus nearly doubled from 4.7% in 1980 to 8.5% in 2014. Most of this increase comes from low- and middle-income countries, and the majority of these patients suffer from type 2 diabetes mellitus (T2DM). T2DM is a disorder characterized by chronic hyperglycemia caused by resistance of body tissues to the effects of insulin [1]. Hyperglycemia causes macrovascular and microvascular complications, which can also affect kidneys. This condition is called diabetic nephropathy (DN) and is present in 20-40% of diabetic patients. DN is defined by decreased glomerular filtration rate and/or elevated levels of urine albumin excretion [2]. Patients with diabetes who suffer from DN are more prone to cardiovascular diseases and have a higher mortality rate than those without DN [3].

Familial clustering of DN can be explained by both genetic and environmental factors, and most likely the combination of the two. Several association studies have identified a strong genetic component to both albuminuria and impaired glomerular filtration rate [4]. Identifying genes that are involved in DN could lead to novel forms of treatment and maybe even prevention of this life-threatening condition.

Sirtuin 1 (SIRT1) is a NAD+-dependent deacetylase, belongs to a group of enzymes called silent information regulators that are expressed in a variety of tissues including kidney, adipose, muscle, liver, and pancreas. It modifies proteins that participate in DNA repair, stress and inflammatory response, and regulation of energy metabolism. SIRT1 promotes lipolysis in white adipose tissue, protects from excessive lipid ­accumulation in skeletal muscle and the liver, and supports insulin secretion [5]. Animal studies show that SIRT1 improves insulin sensitivity and secretion of insulin, making it a strong anti-diabetic agent [6]. It is induced during periods of calorie restriction and indirectly inhibits stress-induced apoptosis [7].

The role of inflammation in diabetes related complications is very well established. SIRT1 suppresses inflammatory pathways in the cell, the expression of pro-inflammatory genes and the secretion of TNF-α, IL-1β, IL-6, p53, FoxO, and other pro-inflammatory molecules [8,9]. A study has specifically linked SIRT1 and chronic renal injury caused by inflammation [10]. SIRT1 also has renoprotective effects, as it offers resistance to inflammation, apoptosis of tubular and glomerular cells and reduces interstitial fibrosis just to name a few [11].

A number of studies showed that SIRT1 and its polymorphisms are involved in visceral obesity, which can lead to type 2 diabetes [5,12-15].

The SIRT1 gene is located on chromosome 10. A study on *SIRT1* polymorphism rs7069102 found that the C allele reduced the risk of visceral obesity in Caucasian males [15]. Studies have linked rs7069102 with genetic susceptibility to chronic obstructive pulmonary disease (COPD) [16], myocardial infarction [17,18], left ventricular myocardial hypertrophy [19], hypertension [20], and early-onset psoriasis [21].

In this study, we investigated the relationship between the *SIRT1* rs7069102 polymorphism and DN in patients with T2DM.

## MATERIALS AND METHODS

### Patients

In our retrospective association study, we included 724 Slovene patients who have had T2DM for at least 10 years. The diagnosis of T2DM and DN was made according to the World Health Organization criteria. We classified the participants in two groups, according to their nephrological diagnosis. The first group was comprised of 301 patients with DN, and the second (control) group was comprised of 423 patients without DN. The exclusion criteria for our study were overt nephropathy, active infection, poor glycemic control (glycated hemoglobin [HbA1c] above 10), significant heart failure (New York Heart Association [NYHA] Classification II-IV), alcoholism, and the presence of other possible causes of renal disease. We used a questionnaire to obtain information about age, sex, duration of T2DM, blood pressure, systolic pressure, diastolic pressure, duration of arterial hypertension (AH), smoking status, and other microvascular complications of T2DM (diabetic retinopathy [DR] and its duration, diabetic foot) and carotid disease. We measured the participants height, weight, and waist circumference. We took blood samples and measured the total cholesterol, high-density lipoprotein (HDL), low-density lipoprotein (LDL), triglycerides, and HbA1c.

All participants provided informed consent before their blood samples were taken and analyzed. Our study was conducted according to the Helsinki declaration and approved by the Slovenian commission for medical ethics. The ethics number for our study was 105/12/2011.

### Biochemical analyses

HbA1c, total cholesterol, HDL, LDL, and triglycerides were determined by standard biochemical methods.

### Genotyping

We extracted genomic DNA from leukocytes in 100 ml of peripheral blood using QIAamp DNA Blood Mini Kit (Qiagen GmbH, Hilden, Germany). We analyzed the rs7069102 polymorphism using StepOne™ (48-well) real-time polymerase chain reaction (PCR) systems (Applied Biosystems by Life Technologies, USA) and TaqMan SNP Genotyping Assay (Applied Biosystems, Foster City, California, USA) according to the instructions of the manufacturers. The volume of the reaction mix was 5 ml. The reaction mix was comprised of TaqMan Universal Master Mix, oligonucleotide primers labeled with VIC/FAM fluorescent dyes, 1.88 μl of purified H_2_O, and 0.5 μl of DNA. The following protocol was used for the amplification of DNA: Step 1, 30 second read of starting fluorescence at 60°C (pre-PCR read); step 2, 10 minutes of starting denaturation at 95°C, followed by 35 cycles of amplification. One cycle consisted of 15 second denaturation at 95°C, followed by 60 second of extension at 60°C; step 3, 30 second read of final fluorescence at 60°C (post-PCR read).

### Statistical analysis

Allele discrimination plots were visualized using StepOne Software version 2.2 (Applied Biosystems, Foster City, California, USA). We used IBM SPSS Statistics for Windows version 21.0 (IBM Corp., Armonk, New York, USA) for statistical analysis. Continuous variables were compared using Student’s t-test or Mann–Whitney’s U test. Chi-square test was used to compare discrete variables. We used logistic regression analysis to analyze the relationship between rs7069102 polymorphism and DN after adjusting for systolic pressure, triglycerides, HbA1c, AH, duration of AH, DR, carotid disease, and diabetic foot. The value of *p* < 0.05 was considered statistically significant. Fisher’s exact test was used to determine the deviation from the Hardy–Weinberg equilibrium (HWE) (http://ihg.gsf.de/).

## RESULTS

Demographic, clinical, and laboratory characteristics of patients are presented in Tables 1 and 2. The groups differed significantly in the following variables: Systolic pressure, AH, duration of AH, triglycerides, value of HbA1c, carotid disease, diabetic foot, and DR. Patients with DN had higher systolic pressure, more frequent and longer lasting AH, higher values of triglycerides and HbA1c, and a higher incidence of carotid disease, diabetic foot, and DR (Tables 1 and 2).

**TABLE 1:**
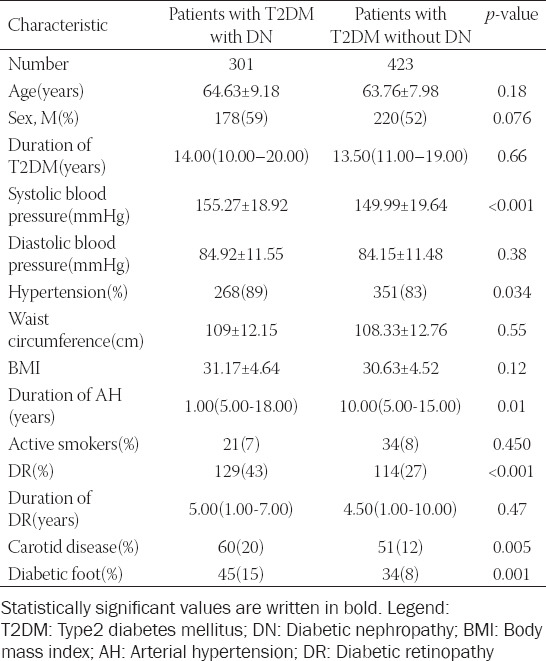
Demographic and clinical characteristics of patients with T2DM with DN and control(patients with T2DM without DN)

**TABLE 2:**
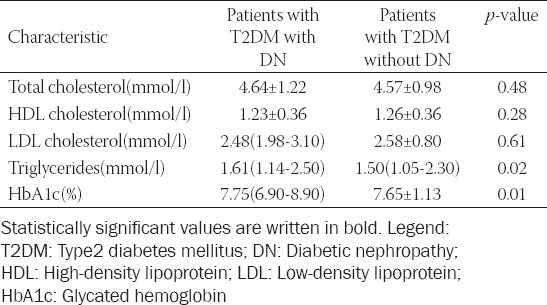
Laboratory characteristics of patients with T2DM with DN and control(patients with T2DM without DN)

Distribution of rs7069102 alleles and genotypes is shown in Table 3. There was a significant difference between the groups in the distribution of C allele as well as the CC and CG genotypes. The C allele, CC, and CG genotypes were more common in the group with DN (Table 3). Both groups were in accordance with the HWE.

**TABLE 3:**
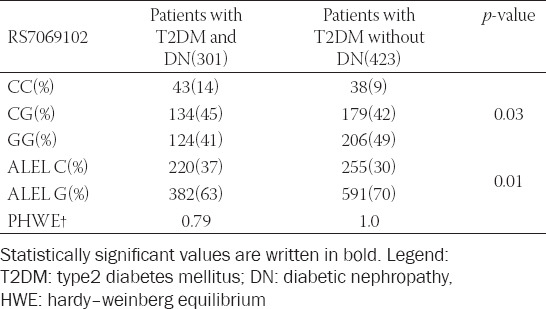
Distribution of rs7069102 polymorphism genotypes and alleles in T2DM patients with DN and without DN

There was no difference in the waist to hip ratios regarding rs7069102 SIRT gene genotypes (*p* = 0.6) (data not shown).

We used logistic regression analysis and adjusted for systolic pressure, AH, duration of AH, triglycerides, value of HbA1c, carotid disease, diabetic foot, and DR to evaluate whether rs7069102 is independently associated with DN (Table 4). We discovered that patients with the CC genotype are significantly more likely to develop DN in comparison with patients with the GG genotype according to the codominant model of inheritance (odds ratio [OR] = 1.94; 95% confidence interval [CI] = 1.09-3.45; *p* = 0.02). Application of the recessive model of inheritance also yielded the same finding, patients with the CC genotype were more likely to develop DN in comparison with the CG and GG genotypes (OR = 2.39; 95% CI = 1.12-5.08; *p* = 0.02). There was no statistically significant correlation between the CG genotype and DN.

**TABLE 4:**
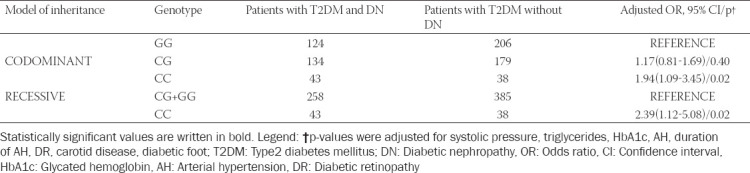
Association of rs7069102 and DN in T2DM patients

## DISCUSSION

In this study, we found an association between the C allele of *SIRT1* rs7069102 polymorphism and DN in patients with T2DM (*p* = 0.01). We used linear regression to statistically determine the relationship between rs7069102 genotypes and DN. We discovered that patients with the CC genotype are more likely to develop DN than patients with other genotypes. Using a codominant model of inheritance, the patients with the CC genotype were 1.94 times more likely to develop DN (*p* = 0.02) than the other genotypes. Moreover, the application of a recessive model of inheritance showed that T2DM patients with the CC genotype are 2.39 times more likely to develop DN than those with the CG or GG genotype (*p* = 0.02; OR: 2.39; 95% CI: 1.12-5.08). The dominant model of inheritance did not show a significant correlation of the CC and CG genotype with DN when compared to the GG genotype.

As previously mentioned, SIRT1 plays a role in many cellular mechanisms that are involved in pathogenesis of DN. A decrease in its activity and/or levels would cause an increase in inflammation, stress-induced apoptosis, and renal fibrosis as well as affect glucose regulation. The rs7069102 polymorphism could affect any or all of the above-mentioned mechanisms, however, further research is needed to determine exactly which process or process’s it affects.

**FIGURE 1:**
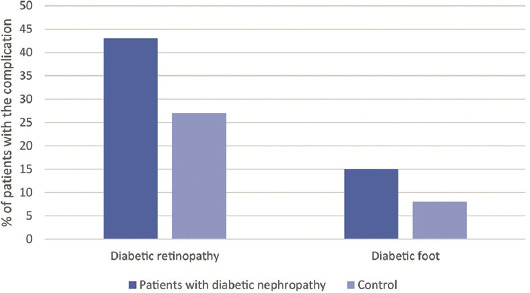
Comparison of the prevalence of diabetic retinopathy (DR) and diabetic foot between patients with diabetic nephropathy (DN) and control. In the group of patients with DN 43% of patients also had DR, whereas only 27% of patients without DN had DR. In the group of patients with DN 15% also had diabetic foot, compared to the 8% who had diabetic foot in the control group.

We also discovered a significant association between DR and DN (*p* < 0.001). In the group of patients with DN, a total of 43% of patients also had DR, whereas only 27% of patients without DN had DR (Figure 1). The strong association between DN and DR has been shown by many studies done on different populations [22-25]. SIRT1 has a protective role in the development of DR; however, there are currently no published studies that investigated the relationship between SIRT1 polymorphisms and DR [26,27].

As of now, ours is the only study that researched the influence of rs7069102 polymorphism on DN in patients with T2DM. However, Maeda *et al*. did research other polymorphisms of the SIRT1 gene and their relationship with DN in Japanese patients with T2DM. Of the 11 *SIRT1* polymorphisms, they investigated, one (rs4746720) showed a significant association with DN. They also found an association between rs4746720 and three other polymorphisms (rs2236319, rs10823108, rs3818292) and combined phenotypes of proteinuria and end stage renal disease (ESRD) [28]. So far, there are two studies that investigated the correlation between *SIRT1* polymorphisms and DN in the Chinese population. Both found a correlation between the rs10823108 polymorphism and DN, thus reinforcing Maeda`s findings [29,30]. Yue *et al*. also described the protective role of another SIRT1 polymorphism rs3818292 against DN [30].

## CONCLUSION

Based on the evidence available, we can say that some polymorphisms of *SIRT1* play a role in the susceptibility of T2DM patients to DN, at least in the Caucasian, Japanese, and Chinese populations. Further research is needed in order to identify more of them, as they could be used as susceptibility markers for DN in T2DM patients. To conclude, we found a significant association between SIRT1 rs7069102 polymorphism and DN in T2DM patients. This implies that *SIRT1* rs7069102 could be used as a marker for susceptibility to DN in T2DM patients.

## References

[1] Roglic G, Varghese C, Riley L, Harvey A, Krug E, Alwan A (2016). Global Report on Diabetes.

[2] Gheith O, Farouk N, Nampoory N, Medhat AH, Torki A (2016). Diabetic kidney disease:World-wide difference of prevalence and risk factors. J Nephropharmacol.

[3] Afkarian M, Sachs MC, Kestenbaum B, Hirsch IB, Tuttle KR, Himmelfarb J (2013). Kidney disease and increased mortality risk in Type 2 diabetes. J Am Soc Nephrol.

[4] Freedman BI, Bostrom M, Daeihagh P, Bowden DW (2007). Genetic factors in diabetic nephropathy. Clin J Am Soc Nephrol.

[5] Kurylowicz A (2016). In search of new therapeutic targets in obesity treatment:Sirtuins. Int J Mol Sci.

[6] Bordone L, Motta MC, Picard F, Robinson A, Jhala US, Apfeld J (2006). Sirt1 regulates insulin secretion by repressing UCP2 in pancreatic b cells. PLoS Biol.

[7] Cohen HY, Miller C, Bitterman KJ, Wall NR, Hekking B, Kessler B (2004). Calorie restriction promotes mammalian cell survival by inducing the SIRT1 deacetylase. Science.

[8] Yoshizaki T, Schenk S, Imamura T, Babendure JL, Sonoda N, Ju Bae E (2010). SIRT1 inhibits inflammatory pathways in macrophages and modulates insulin sensitivity. Am J Physiol Endocrinol Metab.

[9] Wang W, Sun W, Cheng Y, Xu Z, Cai L (2019). Role of sirtuin-1 in diabetic nephropathy. J Mol Med (Berl).

[10] Vachharajani VT, Liu T, Wang X, Hoth JJ, Yoza BK, McCall CE (2016). Sirtuins link inflammation and metabolism. J Immunol Res.

[11] Kitada M, Kume S, Takeda-Watanabe A, Kanasaki K, Koya D (2013). Sirtuins and renal diseases:Relationship with aging and diabetic nephropathy. Clin Sci (Lond).

[12] Clark SJ, Falchi M, Olsson B, Jacobson P, Cauchi S, Balkau B (2012). Association of sirtuin 1 (SIRT1) gene SNPs and transcript expression levels with severe obesity. Obesity (Silver Spring).

[13] Zillikens MC, van Meurs JBJ, Rivadeneira F, Amin N, Hofman A, Oostra BA (2009). SIRT1 genetic variation is related to BMI and risk of obesity. Diabetes.

[14] Weyrich P, Machicao F, Reinhardt J, Machann J, Schick F, Tschritter O (2008). SIRT1 genetic variants associate with the metabolic response of Caucasians to a controlled lifestyle intervention--the TULIP Study. BMC Med Genet.

[15] Peeters AV, Beckers S, Verrijken A, Mertens I, Roevens P, Peeters PJ (2008). Association of SIRT1 gene variation with visceral obesity. Hum Genet.

[16] Kalemci S, Edgunlu TG, Kara M, Turkcu UO, Cetin ES, Zeybek A (2014). Sirtuin gene polymorphisms are associated with chronic obstructive pulmonary disease in patients in Muğla province. Kardiochir Torakochirurgia Pol.

[17] Yamac AH, Uysal O, Ismailoglu Z, Ertürk M, Celikten M, Bacaksiz A (2019). Premature myocardial infarction:Genetic variations in SIRT1 affect disease susceptibility. Cardiol Res Pract.

[18] Cheng J, Cho M, Cen J, Cai M, Xu S, Ma Z (2015). A TagSNP in SIRT1 gene confers susceptibility to myocardial infarction in a Chinese Han population. PLoS One.

[19] Spoto B, Ntounousi E, Testa A, Liakopoulos V, D'Arrigo G, Tripepi G (2018). The sirtuin1 gene associates with left ventricular myocardial hypertrophy and remodeling in two chronic kidney disease cohorts:A longitudinal study. J Hypertens.

[20] Shimoyama Y, Suzuki K, Hamajima N, Niwa T (2011). Sirtuin 1 gene polymorphisms are associated with body fat and blood pressure in Japanese. Transl Res.

[21] Pektas SD, Dogan G, Edgunlu TG, Karakas-Celik S, Ermis E, Tekin NS (2018). The role of forkhead box class O3A and SIRT1 gene variants in early-onset psoriasis. Indian J Dermatol.

[22] Lee WJ, Sobrin L, Lee MJ, Kang MH, Seong M, Cho H (2014). The relationship between diabetic retinopathy and diabetic nephropathy in a population-based study in Korea (KNHANES V-2, 3). Invest Ophthalmol Vis Sci.

[23] Ahmeda MH, Elwalib ES, Awadallac H, Almobarak AO (2017). The relationship between diabetic retinopathy and nephropathy in Sudanese adult with diabetes:Population based study. Diabetes Metab Syndr.

[24] Romero-Aroca P, Sagarra-Alamo R, Baget-Bernaldiz M, Fernández-Ballart J, Méndez-Marin I (2010). Prevalence and relationship between diabetic retinopathy and nephropathy, and its risk factors in the North-East of Spain, a population-based study. Ophthalmic Epidemiol.

[25] Zhang J, Wang Y, Li L, Zhang R, Guo R, Li H (2018). Diabetic retinopathy may predict the renal outcomes of patients with diabetic nephropathy. Ren Fail.

[26] Mishra M, Duraisamy AJ, Kowluru RA (2018). Sirt1:A guardian of the development of diabetic retinopathy. Diabetes.

[27] Karbasforooshan H, Karimi G (2018). The role of SIRT1 in diabetic retinopathy. Biomed Pharmacother.

[28] Maeda S, Koya D, Araki S, Babazono T, Umezono T, Toyoda M (2011). Association between single nucleotide polymorphisms within genes encoding sirtuin families and diabetic nephropathy in Japanese subjects with Type 2 diabetes. Clin Exp Nephrol.

[29] Zhao Y, Wei J, Hou X, Liu H, Guo F, Zhou Y (2017). SIRT1 rs10823108 and FOXO1 rs17446614 responsible for genetic susceptibility to diabetic nephropathy. Sci Rep.

[30] Yue X, Yang Z, Zhang Y, Qin G, Liu F (2018). Correlations between SIRT1 gene polymorphisms and diabetic kidney disease. R Soc Open Sci.

